# RE: What Is the Optimal Treatment for Liver Hydatid Cysts in Emergency and Elective Situations?

**DOI:** 10.5152/tjg.2024.235232

**Published:** 2024-02-01

**Authors:** Serdar Öter, Metin Yalçın

**Affiliations:** 1 Department of Gastroenterological Surgery, Manisa City Hospital, Manisa, Turkey; 2 Department of General Surgery, Antalya Training and Research Hospital, Antalya, Turkey

Dear Editor,

We thank Zerem et al^1^ for their kind words, criticism and contributions to our article titled “Emergency/Elective Surgery and Emergency Percutaneous Interventions in Liver Hydatid Cysts and Their Results”.^2^

We agree with them about percutaneous puncture, aspiration, injection, and re-aspiration (PAIR) should the first choice treatment for hydatid cyst (HC). The PAIR treatment is less invasive and can be applied in more complicated HCs in experienced centers. However, experienced centers for PAIR are not available in every city in Turkey. In addition, even the procurement of the material required for PAIR can sometimes be problematic. Also, in our center, we did not have as much experience as Zerem et al^1^ mentioned at the time when the patients were collected in the PAIR procedure. Therefore, in our article,^2^ PAIR was applied for Type I and Type II HCCs, while laparoscopic or open surgery was preferred in Type III and Type IV.

In addition, persistent biliary fistulas may develop after PAIR treatment in main bile duct-associated HCs. Therefore, it should be kept in mind that surgical drainage and biliary suturing may be required in these patients.

We agree with Zerem et al^1^ that future prospective randomise studies should better determine the place of PAIR method and surgery in the treatment patients with HC.
